# Elevation of antidermatophytic action of mefenamic acid by cobalt ions

**DOI:** 10.4103/0253-7613.71907

**Published:** 2010-12

**Authors:** Ali Abdul Hussein S. AL-Janabi

**Affiliations:** Department of Clinical Laboratory, College of Applicable of Medical Sciences, University of Karbala, Karbala, Iran

**Keywords:** Cobalt, dermatophytes, mefenamic acid

## Abstract

**Objectives:**

To evaluate the antifungal property of mefenamic acid, which is a member of non-steroidal anti-inflammatory drugs (NSAIDs) group.

**Materials and Methods:**

In order to evaluate the antifungal property of mefenamic acid on dermatophytes, it was mixed with cobalt (Co) in culture media. Two species related to two genera of dermatophytes were tested for their susceptibility to mefenamic acid and its complex with Co by using colony diameter measurement method.

**Results:**

The inhibitory action of mefenamic acid on fungal strains was increased in the presence of Co. *Epidermophyton floccosum* showed more susceptibly to either mefenamic acid or its complex with Co than *Trichophyton mentagrophytes* variant *mentagrophytes*.

**Conclusions:**

Mefenamic acid showed potential ability to prevent growth of dermatophytes. This ability increased due to the presence of Co.

## Introduction

Mefenamic acid has pharmacologic actions similar to those of other non-steroidal anti-inflammatory drugs (NSAIDs). The drug exhibits anti-inflammatory, analgesic, and antipyretic activity.[1] Large amounts of mefenamic acid are consumed every year due to these therapeutic properties, especially during summer.[2]

Mefenamic acid is demonstrated to have antibacterial effects based on the susceptibility of seven pathogenic bacteria to this agent.[3] However, many fungal species showed resistance to or no affect of mefenamic acid. *Candida albicans* is affected by this drug. It is considered to be an important opportunistic pathogenic fungus.[4] Cobalt (Co) is considered as one of important metals for fungal development through its association with Ni transporter system into the cells.[5] On the other hand, Co may have antimicrobial activities, especially when it complexes with many synthetic chemical structures. These complexes could exhibit a moderate inhibitory effect on many microbial proliferations.[6] Dermatophytes are special group of pathogenic fungi that can infect human skin and cause lesions in cutaneous layer.[7] They are not influenced by the presence of mefenamic acid in their media (data not shown), while its complexes may have such influences. *Epidermophyton floccosum*, one species of dermatophytes, is found to be inhibited at concentrations ranging from 6 to 50 *μ*g/mL of Co complex (thiophene).[8] However, Edrissi *et al*.[9] illustrated that mefenamic acid can react with Co(II) ions to form highly stable complex at ambient temperature. Thus, mixing of mefenamic acid with Co in culture media was studied to detect any possible effects of such mixture on dermatophytes.

## Materials and Methods

### Organisms

Dermatophytes were clinically isolated from a old infected with dermatophytoses (Tinea corporis) at AL-Hussein General Hospital of Karbala province in February 2009. Skin scales of fungal lesion were cultured on Sabouraud’s glucose agar comprising of the following components: glucose 20 g, peptone 10 g, agar 15 g, chloramphenicol 0.05 g, cycloheximide 0.5 g, and 1000 mL of distilled water. Cultures were incubated at 28°C for 2 weeks. Grown fungi isolated from the same lesion were diagnosed according to criteria reported by Rippon[10] and Emmons *et al*.[11] which also include biochemical tests.

The isolated strains were related to two different genera (*Trichophyton* and *Epidermophyton*): *Trichophyton mentagrophytes* variant *mentagrophytes* and *E. floccosum*.

### Chemical agents

Mefenamic acid was supplied from Brown and Burk Pharmaceutical Limited, Sipcot, Hosur India. Cobalt nitrate (Co(II)) was purchased from Hopkin and Williams Ltd., Chadwell Heath, ESSEX, England. Griseofulvin was supplied by Arabic Drug Industry (Baghdad, Iraq).

### Antidermatophytic assay

Colony diameter method recorded by *Kücüc and Kivan*[12] was used. Various concentrations of mefenamic acid (0.156, 0.3125, 0.625, 1.25, and 2.5 mg/mL) were mixed with melted Sabouraud’s glucose agar containing 0.5% dimethyl sulfoxide (DMSO). Co of 0.5 mg/mL was mixed with each previous concentration of mefenamic acid. Then, prepared media were poured in Sterile Petri dishes. In addition to free media as control, either Co, mefenamic acid, or griseofulvin (10 *μ*g/mL) was used as control after mixed with prepared media. A disk (9 mm) of old grown fungi (at 28°C for 1 week) was inoculated on the center of culture media. Plates were incubated at 28°C for 1 week. Perpendicular colony diameters (mm) of grown strains were measured and percentage inhibition calculated according to the formula:

$$\mathrm{Percentage}\;\mathrm{inhibition}\;=\;\frac{(C\;-\;T)\;\times \;100}{C}$$

where *C* is colony diameter (mm) of the control and *T* is colony diameter (mm) of the test plate.

## Results

**Figure 1:**
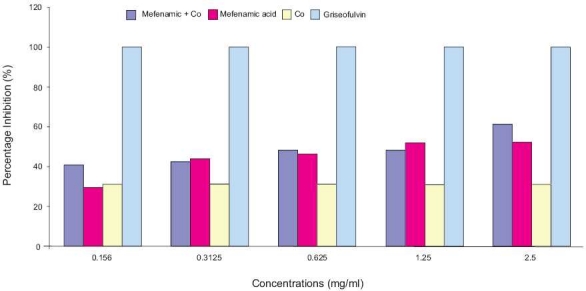
Effect of mefenamic acid, its complex with Co, and griseofulvin (10 μg/mL) on *T. mentagrophytes* variant *mentagrophytes*.

**Figure 2:**
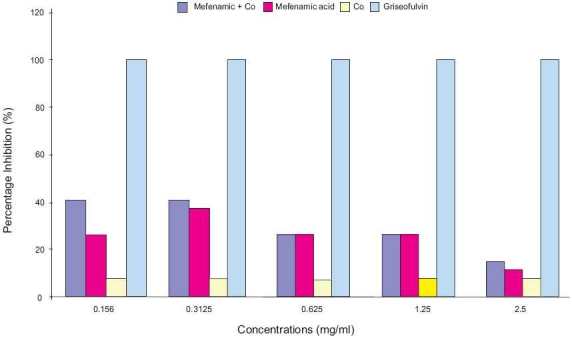
Effect of mefenamic acid, its complex with Co, and griseofulvin (10 μg/mL) on *E. floccosum*.

Activity of mefenamic acid complex with Co was investigated against dermatophytes. Compared to control (free media) and media containing Co only, incorporation of various concentrations of mefenamic acid in culture media showed decrease in fungal colony diameter of isolated strains. These results are indicated by high percentage inhibition of grown strains [Figures 1 and 2], which showed an increase in the antidermatophytic activity of mefenamic acid due to the presence of Co ions.

*E. floccosum* exhibited more susceptibility to mefenamic acid and its complex with Co (high ratio of inhibition) than *T. mentagrophytes* variant *mentagrophytes* [Figure 2]. However, neither mefenamic acid alone or with Co exceeded the effect of standard antidermatophytic agent (griseofulvin) [Figures 1 and 2].

## Discussion

Wide spread dermatophyte infections among people over the world and failure of most drugs to cure such infections are a stimulus for continuous search for new drugs effective against dermatophytes. Thus, many chemical compounds are being tested for determining their activity on dermatophytes.

Cobalt has the ability to enhance the action of some antimicrobial drugs. Antibacterial action of ampicillin and chloramphenicol was increased when mixed with Co ions.[13] This encourages us to test the interaction of Co and mefenamic acid against dermatophytes. Mefenamic acid was chosen from NSAID group n the basis of prior reported interaction of this agent with Co ions.[9] Thus, any weakness in antifungal action of mefenamic acid could be diminished through mixing with such type of metal. Furthermore, low inhibitory action of Co had been increased due to mix with mefenamic acid. Therefore, enhancement the action of mefenamic acid and Co on dermatophytes could be proposed.

Like other NSAIDs, mefenamic acid shows its therapeutic activity through inhibition of prostaglandin synthesis in body tissues. This action is performed by inhibition of cyclooxygenase (COX) enzyme which is important for prostaglandin synthesis.[14] Dermatophytes are proven to have the ability to produce prostaglandin which is responsible for chronic fungal colonization.[15] Furthermore, COX enzyme was also demonstrated to be present in fungal cells.[16] Thus, mefenamic acid may act against dermatophytes through inhibition of COX enzyme. Co may play a role in entry of mefenamic acid into cell through cell membrane of dermatophytes in mechanism similar to that with Ni transport.[5] Therefore, mefenamic acid can easily access its site of action in fungal cells. Moreover, an important property of mefenamic acid is its inability to dissolve in water.[17] Thus, interaction of mefenamic acid with Co may increase solubility rate of this member of NSAIDs.

*E. floccosum* exhibited more susceptibility to mefenamic acid and its complex. This may be related to the absence of microconidia in this species compared with other species of dermatophytes.[10,11] Thus, *E. floccosum* has less efficiency in reduction of the toxic action of mefenamic acid due to lower fungal mass. *T. mentagrophytes* has a heavy density of fungal elements that is composed of macroconidia, microconidia, and fungal hyphae.

Increasing the concentrations of mefenamic acid led to enhanced inhibitory action of this agent on two isolated dermatophytes either added alone or with Co ions in culture media. However, low concentrations of mefenamic acid complex continued to show high degree of inhibition than Co.

In conclusion, mefenamic acid showed ability to prevent dermatophytes. This ability was enhanced by the presence of Co. High percentage inhibition was noted distinctly after mixing of mefenamic acid with Co. Furthermore, *E. floccosum* exhibited more susceptibility to mefenamic acid than *T. mentagrophytes*.

## References

[1] McEvoy GK, Litvak K, Welsh OH, Dewey DR, Fong PA, Douglas PM (1993). AHFS drug information. Am Soc Hosp Pharm.

[2] Takao Y, Shimazu M, Fukuda M, Ishibashi H, Nagae M, Kohra S (2008). Seasonal and diurnal fluctuation in the concentrations of pharmaceuticals and personal care products (PPCPs) in residential sewage water. J Health Sci.

[3] AL-Janabi AA (2009). Comparison of the disc diffusion assay with spectrophotometer technique for antibacterial activity of diclofenac sodium, indomethacin and mefenamic acid. Asian J Pharm.

[4] Kruszewska H, Zareba T, Tyski S (2006). Estimation of antimicrobial activity of selected non-antibiotic products. Acta Pol Pharm.

[5] Zhang Y, Rodionov DA, Gelfand MS, Gladyshev VN (2009). Comparative genomic analyses of nickel, cobalt and vitamin B12 utilization. BMC Genomics.

[6] Rodríguez-Argüelles MC, Mosquera-Vázquez S, Tourón-Touceda P, Sanmartín-Matalobos J, García-Deibe AM, Belicchi-Ferrari M (2007). Complexes of 2- thiophenecarbonyl and isonicotinoyl hydrazones of 3-(N-methyl)isatin.: A study of their antimicrobial activity. J Inorg Biochem.

[7] Hainer BL (2003). Dermatophyte infections. Am Fam Physician.

[8] Rodríguez-Argüelles MC, López-Silva EC, Sanmartín J, Bacchi A, Pelizzi C, Zani F (2004). Cobalt and nikel complexes of versatile imidazole-and pyrrole-2-carbaldehyde thiosemicarbazones. Synthesis, characterization and antimicrobial activity. Inorganica Chimica Acta.

[9] Edrissi M, Asl NR, Madjidi B (2008). Interaction of mefenamic acid with cobalt (II) ions in aqeous media: Evaluation via classic and response surface methods. Turk J Chem.

[10] Rippon JW (1988). Medical mycology.

[11] Emmons CW, Binford CH, Utz JP (1970). Medical mycology.

[12] Kücüc G, Kivan M (2003). Isolation of *Trichoderma* Spp. and determination of their antifungal, biochemical and physiological features. Turk J Biol.

[13] Ogunniran KO, Ajanaku KO, James OO, Ajani OO, Adekoya JA, Nwinyi OC (2008). Synthesis, charactererization, antimicrobial activity and toxicology study of some metal complexes of mixed antibiotics. Afr J Pure Appl Chem.

[14] Feldman M, McMahon AT (2000). Do cyclooxygenase-2 inhibitors provide benefits similar to those of traditional nonsteroidal anti-inflammatory drugs, with less gastrointestinal toxicity. Ann Intern Med.

[15] Noverr MC, Toews GB, Huffnagle GB (2002). Production of prostaglandins and leukotrienes by pathogenic fungi. Infect Immun.

[16] Tsitsigiannis DI, Bok JW, Andes D, Nielsen KF, Frisvad JC, Keller NP (2005). Aspergillus cyclooxygenase-like enzyme are associated with prostaglandin production and virulence. Infect Immun.

[17] Derle DV, Bele M, Kasliwal N (2008). *In vitro* and *in vivo* evaluation of mefenamic acid and its complexes with β-cyclodexin and HP- β-cyclodexin. Asian J Pharm.

